# Reduced use of phosphorus and water in sequential dark fermentation and anaerobic digestion of wheat straw and the application of ensiled steam-pretreated lucerne as a macronutrient provider in anaerobic digestion

**DOI:** 10.1186/s13068-018-1280-z

**Published:** 2018-10-11

**Authors:** Eoin Byrne, Krisztina Kovacs, Ed W. J. van Niel, Karin Willquist, Sven-Erik Svensson, Emma Kreuger

**Affiliations:** 1 Division of Applied Microbiology, Dept. of Chemistry, Lund University, PO Box 124, 221 00 Lund, Sweden; 2 Dept. of Chemical Engineering, Lund University, PO Box 124, 221 00 Lund, Sweden; 3 RISE, Forskningsbyn Ideon Scheelevägen 27, 223 70 Lund, Sweden; 4 Dept. of Biosystems and Technology, Swedish University of Agricultural Sciences, PO Box 103, 230 53 Alnarp, Sweden; 5 Division of Biotechnology, Dept. of Chemistry, Lund University, PO Box 124, 221 00 Lund, Sweden

**Keywords:** Intercropping, Energy crops, Alfalfa, *Caldicellulosirupto*r, Thermophilic hydrogen production, Osmotolerance, Biogas, Co-substrate, UASB, Biofuel

## Abstract

**Background:**

Current EU directives demand increased use of renewable fuels in the transportation sector but restrict governmental support for production of biofuels produced from crops. The use of intercropped lucerne and wheat may comply with the directives. In the current study, the combination of ensiled lucerne (*Medicago sativa* L.) and wheat straw as substrate for hydrogen and methane production was investigated. Steam-pretreated and enzymatically hydrolysed wheat straw [WSH, 76% of total chemical oxygen demand (COD)] and ensiled lucerne (LH, 24% of total COD) were used for sequential hydrogen production through dark fermentation and methane production through anaerobic digestion and directly for anaerobic digestion. Synthetic co-cultures of extreme thermophilic *Caldicellulosiruptor* species adapted to elevated osmolalities were used for dark fermentation.

**Results:**

Based on 6 tested steam pretreatment conditions, 5 min at 200 °C was chosen for the ensiled lucerne. The same conditions as applied for wheat straw (10 min at 200 °C with 1% acetic acid) would give similar sugar yields. Volumetric hydrogen productivities of 6.7 and 4.3 mmol/L/h and hydrogen yields of 1.9 and 1.8 mol/mol hexose were observed using WSH and the combination of WSH and LH, respectively, which were relatively low compared to those of the wild-type strains. The combinations of WSH plus LH and the effluent from dark fermentation of WSH plus LH were efficiently converted to methane in anaerobic digestion with COD removal of 85–89% at organic loading rates of COD 5.4 and 8.5 g/L/day, respectively, in UASB reactors. The nutrients in the combined hydrolysates could support this conversion.

**Conclusions:**

This study demonstrates the possibility of reducing the water addition to WSH by 26% and the phosphorus addition by 80% in dark fermentation with *Caldicellulosiruptor* species, compared to previous reports. WSH and combined WSH and LH were well tolerated by osmotolerant co-cultures. The yield was not significantly different when using defined media or hydrolysates with the same concentrations of sugars. However, the sugar concentration was negatively correlated with the hydrogen yield when comparing the results to previous reports. Hydrolysates and effluents from dark fermentation can be efficiently converted to methane. Lucerne can serve as macronutrient provider in anaerobic digestion. Intercropping with wheat is promising.

**Electronic supplementary material:**

The online version of this article (10.1186/s13068-018-1280-z) contains supplementary material, which is available to authorized users.

## Background

According to the EU Renewable Energy Directive 2009/28/EC, 10% of the energy used within the transportation sector in the EU should be renewable in 2020 [1]. In an amendment, it is stated that the contribution of biofuels from crops rich in oil, sugar or starch, as well as energy crops causing high indirect land use change, should be restricted to 7% of the energy used in the transportation sector by 2020 [2]. In 2015, only 6.7% of the energy used in the transportation sector in the EU was renewable, and most was of the type that should be phased out [3]. To increase the production of biofuel despite these restrictions, the EU is promoting research and development of biofuels (transportation fuels) from feedstock that does not compete with food production [1, 2].

Methane and hydrogen are interesting energy carriers with respect to meeting the EU’s 10% goal of renewable fuel in domestic transportation. Methane can be produced from organic waste and by processing residues through anaerobic digestion, and can be converted into other fuels, using processes with low environmental impact [4, 5]. Hydrogen production through dark fermentation is less well developed, but this gas is considered a promising energy carrier since its combustion produces zero carbon emissions [6]. Research in the production of these energy carriers is further motivated by their use in electricity generation and chemical synthesis, e.g. ammonia [7] or oil and fat hydrogenation [8]. Another advantage of anaerobic digestion is that the residues, i.e. the biodigestates, are widely applied as fertilizers in agriculture [9, 10].

Wheat straw is one of the most abundant crop residues in the world and, thus, an interesting substrate for biofuel production [11]. It is one of the raw materials of choice in the first commercial cellulosic ethanol plant, which was opened in Crescentino, Italy in 2013 [12]. The effluent from ethanol production contains residual organic material that has been successfully converted into methane in up-flow anaerobic sludge blanket (UASB) reactors [13]. Using direct anaerobic digestion, simply chopped wheat straw can give a relatively high methane yield with long retention times [310 L per kg volatile solids (VS) after 127 days] [14]. Steam pretreatment has been shown to accelerate degradation [14] resulting in a 39% increase in methane yield after 31 days of digestion in a batch test (methane 250 L per kg VS compared to 180 L per kg VS using untreated chopped straw) [14]. It has also been shown to be relatively energy and cost efficient pretreatment method [15, 16]. Except from increasing the conversion rate, steam pretreatment and enzymatic hydrolysis have the potential to reduce reactor sizes and, thereby, processing costs, due to the possibility of applying higher organic loading rates in anaerobic digestion when using liquid instead of solid substrates [17]. A high degree of conversion of solubilised sugars has been demonstrated for steam-pretreated and enzymatically hydrolysed wheat straw in sequential thermophilic dark fermentation, with *Caldicellulosiruptor saccharolyticus*, in stirred tank reactors, and mesophilic anaerobic digestion (with mixed microbial culture) in UASB reactors [14, 18]. The main by-product of *C. saccharolyticus* is acetate, which was readily converted, together with many non-metabolised compounds, to methane in the anaerobic digestion process.

The extremely thermophilic *Caldicellulosiruptor* species exhibit hydrogen yields near the theoretical thermodynamic maximum for both hexose sugars (4 mol H_2_ per mol) and pentose sugars (3.33 mol H_2_ per mol). They are also relatively tolerant to high hydrogen partial pressures up to 67 kPa [18, 19]. In the study by [18], a hydrogen productivity of 8.69 mmol/L/h was achieved from 10 times diluted wheat straw hydrolysate (WSH). *C. saccharolyticus* is, however, unable to grow in concentrated hydrolysates due to its osmosensitivity. Strains of *C. saccharolyticus* species that tolerate higher osmolality have recently been developed through adaptive laboratory evolution for use in higher concentration substrates. They have been shown to tolerate between 50 and 80 g/L sugar, compared to 10 g/L sugar for the parental strain [20, 21]. Increased substrate concentration can reduce water and energy demands in dark fermentation considerably [22]. In addition, synthetic co-cultures of *Caldicellulosiruptor* species have displayed better performance than pure cultures [23]. However, it remains to be determined whether these strains also tolerate more highly concentrated hydrolysates from plant residues.

One drawback of using wheat straw in dark fermentation and anaerobic digestion is the low content of many crucial nutrients and, thus, nutrient supplements are needed. However, the use of nutrients derived from mining (e.g. phosphorus) and ammonium produced with help of fossil fuels is not a sustainable practice [7, 24]. In addition, nutrients such as yeast extract and phosphates contribute substantially to the production costs, making this process uncompetitive with fossil-based fuel production [25]. Although macronutrients can be provided by the addition of co-substrates such as certain industrial wastes, organic fractions of municipal household waste, or manure, the availability varies geographically [26]. Alternatively, a lack of co-substrate can be mitigated by the cultivation of a nutrient-rich crop for use as a co-substrate.

Lucerne (*Medicago sativa* L.), also known as alfalfa, is a widely cultivated legume and has several favourable characteristics in agricultural production [27, 28]. Lucerne is cultivated on approx. 30 million hectares worldwide, of which 25% (7.12 million hectares) is located in Europe [29]. Like other legumes, lucerne can form symbiosis with nitrogen-fixing microorganisms and thereby supply nitrogen. Furthermore, nitrogen and other nutrients can be cycled by fertilising the soil with the residues of the anaerobic digestion process and, thereby, reduce the input of industrially synthesised or mined minerals [30].

Legume intercropping with wheat in organic cultivation has been shown to increase the grain yield in subsequent maize cultivation [31]. Further, intercropping of legumes in organic winter wheat was found not to influence the grain yield. However, in one-third of cases it did reduce the wheat grain protein content [32]. In the same trials, intercropping was found to reduce the density of weeds [33]. Spring fertilisation with organic fertilizer was recently reported to decrease the legume biomass yield, but improve the grain yield and protein content [34]. Although the legume was left on field in the above-mentioned studies, it can also be harvested together with the wheat straw some weeks after the wheat grain harvest, for use as a substrate for dark fermentation and anaerobic digestion. After fermentation, the residue can be used as fertilizer. Intercropping has the potential to comply with the land use regulations in the EU directives [1, 2].

Anaerobic digestion of lucerne resulted in a methane yield of 280 to 430 L per kg VS [27]. These yields are in the same range as the methane yields from maize, which is one of the most widely used energy crops in commercial anaerobic digestion and used without other pretreatment than chopping and ensiling [35]. Therefore, it is uncertain if it is economically advantageous to pretreat pure lucerne prior to anaerobic digestion. However, in dark fermentation using co-cultures of only a few species, many of the diverse compounds may not be degraded [36] and, therefore, part of the sugar polymers might not be accessible without pretreatment.

Some groups have investigated the pretreatment of lucerne for ethanol production, but not for the production of other fuels. Ambient-temperature acid pretreatment followed by ensiling [37] dilute-acid pretreatment [38, 39], ammonia pretreatment [39, 40], wet explosion [41], hydrothermal pretreatment [42] and steam pretreatment without a catalyst [40] have been evaluated in attempts to improve the degradability of lucerne prior to ethanol production.

Compounds synthesised from fossil fuels or mined from non-renewable sources are commonly used not only as nutrient supplements and as buffering agents, but also as mineral acid catalysts in steam pretreatment. Joelsson et al. [43] showed that mineral acids could be replaced by acetic acid in steam pretreatment of wheat straw for ethanol and biogas production, without decreasing the biofuel yield. Another option is to use ensiled raw materials [44], which already contain organic acids, such as lactic acid and acetic acid. This will reduce the cost of chemicals in the process. Since acetic acid is produced during dark fermentation by *Caldicellulosiruptor* spp. and can be degraded in anaerobic digestion, the application of acetic acid as a catalyst opens the possibility for future process integration with internal production, use and degradation.

**Fig. 1:**
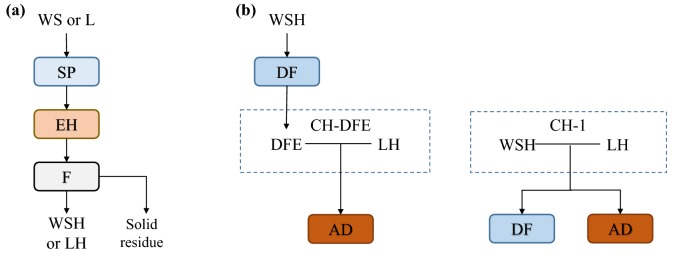
Flow chart illustrating **a** hydrolysis of plant material and **b** use of hydrolysates in dark fermentation and anaerobic digestion. *AD* Anaerobic digestion, *CH-DFE* combined hydrolysate-dark fermentation effluent, *DF* dark fermentation, *EH* enzymatic hydrolysis, *F* fractionation, *L* lucerne, *LH* lucerne hydrolysate, *SP* steam pretreatment, *WS* wheat straw, *WSH* wheat straw hydrolysate

In the current study, wheat straw and ensiled lucerne were steam pretreated and hydrolysed using enzymes. After the enzymatic hydrolysis (EH), the materials were filtered to obtain the wheat straw hydrolysate (WSH) and the lucerne hydrolysate (LH) (Fig. 1a). Wheat straw was steam pretreated with acetic acid as catalyst and lucerne both with and without acetic acid as a catalyst, followed by enzymatic hydrolysis (EH). Lucerne cultivated in pure culture was used, because of the unavailability of lucerne intercropped with wheat and the advantages of optimising conditions for pure substrates rather than mixtures (Fig. 1).

The WSH was subjected to dark fermentation using a synthetic co-culture of two osmotolerant strains in a chemostat. The effluent from dark fermentation was then mixed with lucerne hydrolysate (LH) and subjected to anaerobic digestion in UASB reactors. Combined WSH and LH were also investigated separately for dark fermentation and for anaerobic digestion (Fig. 1b).

## Methods

### Raw materials

Straw of winter wheat was provided by Johan Håkansson Lantbruksprodukter, Lunnarp, Sweden. Dry matter (DM) was 90% of wet weight (ww) and volatile solids (VS) 94% of DM. The raw material was milled using a knife mill (Retsch GmbH, Haan, Germany) and sieved with a vibrating Vibrodynamics screening unit (SWG Process Engineering Ltd., England) to obtain 2–10-mm pieces and then stored at 4 °C before steam pretreatment. The contents of glucan (29.3%), xylan (21.6%), arabinan (3.2%), lignin (27.5%), ash (1–2%) and extractives (15%) of wheat straw from the same straw bale have been determined previously [45].

Ensiled lucerne (cultivar Nexus from Lantmännen Lantbruk, Malmö, Sweden) with 35.5% DM was provided by Naturpollen Axet AB, Höganäs, Sweden. The lucerne was sown in April 2014 in autumn-sown rye (the rye was harvested in August in 2014) at latitude 56.187 and longitude 12.628. The lucerne was fertilised with organic manure in spring 2015 (20 kg N, 10 kg P and 65 kg K per hectare) and harvested twice. The second harvest (used in the current study) was done at early flowering stage in August 2015. The crop was air dried on the field for 24 h prior baling and ensiled, without additives, for 9 months. The wet silage was milled in a garden shredder (Turbine Cut System, AXT 25TC, Robert Bosch GmbH, Germany) and then stored at − 20 °C before pretreatment.

The composition of ensiled lucerne was analysed to determine the contents of structural carbohydrates, lignin, protein, soluble sugars and organic acids according to the National Renewable Energy Laboratory (NREL) procedures [46, 47]. Ensiled lucerne contained (% of DM) 13.3% glucose, 8.4% xylose, 3.1% galactose, 2.6% arabinose, 7.1% lignin, 21.9% protein, 2.1% acetic acid, 5.2% lactic acid, and 40.3% extractives, where the sugars were expressed in monomer equivalent.

### Preparation of wheat straw hydrolysate

The wheat straw was steam pretreated as described by Bondesson and Galbe [45]. Briefly, the raw material was soaked overnight in an aqueous solution containing 1 wt% acetic acid at room temperature. The total weight of liquid was 20 times that of the dry wheat straw. The soaked material was dewatered in a 25-L automatic filter press (Tinkturenpressen HP25 M, Fischer Maschinenfabrik GmbH, Germany) to a DM content of approximately 50%. The impregnated wheat straw was steam pretreated in a 10-L unit as described previously [48], in which the equivalent of 400 g dry weight straw was loaded into the reactor at a time. The temperature during pretreatment was 190 °C and the residence time was 10 min [45].

To prepare the WSH, the whole steam-pretreated wheat straw slurry was subjected to EH in a 60-L vessel equipped with a mechanical impeller. The total working weight was 40 kg and the initial water-insoluble solids (WIS) content was 10%. The temperature was set to 45 °C and the pH was maintained at 4.8 by the addition of 50 wt% NaOH solution. The commercial enzyme preparation Cellic Ctec2 (Novozymes A/S, Bagsværd, Denmark) was used at an enzyme loading of 10 Filter Paper Units (FPU) per g WIS. After 72 h of hydrolysis, the unhydrolysed solid residue was separated from the hydrolysate by vacuum filtration through a Whatman grade 5 qualitative filter paper (Sigma-Aldrich).

### Steam pretreatment and enzymatic hydrolysis of ensiled lucerne

The ensiled lucerne was subjected to steam pretreatment with or without the addition of acetic acid as catalyst. In the case of acid impregnation, the ensiled lucerne was pressed in an automatic filter press to a DM of about 50%, as described above for the WSH. Acetic acid (1 wt%, based on the liquid content of ensiled lucerne) was added to the filtrate, which was then sprayed over the pressed lucerne through a nozzle creating a mist. The material was agitated in a cement mixer to ensure even distribution of the acid.

The ensiled lucerne (impregnated or not) was loaded into the 10-L steam pretreatment unit at 400 g DM at a time, as described above for the WSH. Six different pretreatment conditions were evaluated (Table 1).

**Table 1:** Conditions used in the steam pretreatment of ensiled lucerne

Pretreatment	Catalyst	Temp (°C)	Time (min)
1	No catalyst	200	5
2	No catalyst	200	10
3	No catalyst	210	5
4	1 wt% acetic acid	190	10
5	1 wt% acetic acid	200	5
6	1 wt% acetic acid	210	5

After pretreatment, to determine the composition of the pretreated materials, approximately 100 g of each material was separated into a solid and a liquid fraction as described above for WSH. The solid fraction was analysed to determine the structural carbohydrates, lignin and ash, and the contents of oligomeric and monomeric sugars, organic acids and sugar degradation products were determined in the liquid fraction according to NREL protocols [47, 49].

To evaluate the effect of pretreatment at different conditions, steam-pretreated lucerne samples were subjected to EH in 500-mL sealed flasks using an initial WIS content of 5% and a total weight of 300 g. Cellic Ctec2 was used at an enzyme loading of 10 FPU/g WIS. The flasks were incubated on a rotary shaker for 72 h at 45 °C, pH 4.8 and 180 rpm. After the EH, the sugar contents of the hydrolysates were determined using HPLC and the sugar yields in g sugar per g DM ensiled lucerne were calculated.

To prepare a larger batch of LH for dark fermentation and anaerobic digestion, the ensiled lucerne was steam pretreated in 400 g DM batches at 200 °C for 10 min without the addition of acetic acid. The pretreated material was then subjected to EH in a 60-L vessel, under the same conditions as described for the WSH, except that the initial WIS content of the steam-pretreated lucerne was 7.5%.

### Strain and culture medium and medium development for dark fermentation

The wild-type strain *C. saccharolyticus* DSM 8903 was obtained from the Deutsche Sammlung von Mikroorganismen und Zellkulturen (DSMZ; Braunschweig, Germany). Osmotolerant strains, *C. saccharolyticus* CS50 [21] and *C. owensensis* CO80 [20], were developed via adaptive laboratory evolution. *C. owensensis* CO80 was developed as per Pawar [21] and involved the cultivation of C. *owensensis* DSM 13100 in a medium containing 10 g/L of glucose and then repeatedly sub-cultivated in media containing 10 g/L of glucose more when the generation time for each strain was less than 0.4 h^−1^ and OD was above 0.3 [20]. This sequential increase of glucose concentration was continued until 80 g/L as no growth was observed at higher glucose concentrations [20] Sub-cultivations were carried out in 250 mL serum flasks with 50 mL modified DSM 640 [50] with the addition of 50 mM HEPES.

Glucose was used as the carbon and energy source at concentrations of 10 g/L and 30 g/L, for the wild-type and osmotolerant strains, respectively. A 1000× modified SL-10 solution and a 1000× vitamin solution were prepared as described previously [36, 51].

Fermentors containing a defined medium, as described previously [36], were autoclaved. Anoxic solutions of glucose (10 g/L), cysteine HCl·H_2_O (1 g/L) and MgCl_2_·6H_2_O (0.4 g/L) were prepared independently and were added to the fermentor before inoculation. *C. saccharolyticus* DSM 8903 was cultivated in batch fermentors and the contents of each fermentor were removed when the optical density reached 1, and were then rapidly cooled on ice. The cell solutions were centrifuged at 4000 rpm at 4 °C for 10 min and washed twice with 0.9% NaCl solution. The cell biomass was dried at 60 °C for 24 h, and the elemental composition was quantified (Additional file 1: Table S1) and used to determine the concentration of each element in the 500× EB-1 trace element solution that would allow the formation of up to 3 g/L cell mass. The 500× EB-1 trace element solution consisted of 1500 mg/L FeCl_2_·4H_2_O, 350 mg/L ZnCl_2_, 50 mg/L MnCl_2_·4H_2_O, 3 mg/L H_3_BO_3_, 45 mg/L CoCl_2_·2H_2_O, 10 mg/L CuCl_2_·6H_2_O, 6 mg/L NiCl_2_·6H_2_O, 7.5 mg/L Na_2_WO_4_, 10 mg/L Na_2_SeO_3_·5H_2_O and 12.8 g/L nitrilotriacetate.

### Fermentor set-up for dark fermentation

Synthetic co-cultures of *C. owensensis* strain CO80 and *C. saccharolyticus* strain CS50 were grown in a continuous culture on: (i) 33.3% ww WSH and 66.7% deionised water (WSH-1), (ii) 25.7% ww WSH and 7.7% ww LH and 66.6% deionised water (Combined hydrolysate-1; CH-1), (iii) a defined medium containing 19 g/L glucose, 8.2 g/L xylose and 0.59 g/L arabinose (EB-1), or (iv) defined medium containing the same sugar mixture as EB-1 (modified DSM 640 medium). Chemical oxygen demand (COD) and nutrient content of WSH-1 and CH-1 are presented in Additional file 1: Table S1. Each hydrolysate solution was supplemented with 80 mg/L MgCl_2_·6H_2_O, 900 mg/L NH_4_Cl, 150 mg/L KH_2_PO_4_, 300 mg/L K_2_HPO_4_, 200 mg/L cysteine·HCl, 2 mL/L 500× EB-1trace element solution and 1 mL/L 1000× vitamin solution.

A working volume of 1 L was used for all cultivations unless otherwise stated. The pH was maintained at 6.5 ± 0.1 at 70 °C by automatic titration with 4 M NaOH, and the temperature was maintained at 70 ± 1 °C using a thermostat. Stirring was maintained at 250 rpm and nitrogen was sparged through the medium at a rate of 6 L/h. A water-cooled condenser was used (4 °C) at the gas outlet to prevent evaporation of the medium. During each cultivation, samples were collected at regular intervals to monitor the optical density. The supernatant of each sample was collected and stored at − 20 °C for further quantification of sugars, organic acids, furfural and HMF. Gas samples were taken from the fermentor headspace to quantify the H_2_ and CO_2_ concentrations.

### Cultivation for dark fermentation

Prior to continuous mode, the fermentors were started in batch mode using a modified DSM 640 medium [36] with the addition of 30 g/L glucose. Each cultivation used a mixture of LH and WSH and was run in duplicate as a continuous culture at an HRT of 20 h and a working volume of 1 L. Cultivation with WSH-1 was run initially in fed batch mode for 6 h by introducing 75 mL/h WSH-1, and allowing the volume to increase to 1.5 L, after which the fermentor was operated in continuous mode at an HRT of 20 h and a working volume of 1.5 L.

### Anaerobic digestion

Anaerobic digestion was performed in four water-jacketed UASB reactors (0.86 L working volume) as described previously [14], with the addition of an extra gas outlet to each reactor to avoid pressure build-up in the reactor headspace, leading to a 0.06 L increase in the working volume. The up-flow liquid circulation rate corresponded to the volume of the reactor’s cross-sectional area × 1 m/h. Micronutrients and iron (see below) were added directly to each reactor twice per week. At start-up, the temperature was set to 32 °C, after which it was increased by 1 °C/day to 37 °C, and subsequently it was kept constant throughout the experiment.

Approximately, 500 mL substrate was fed into the anaerobic digestion substrate vessel (total volume of 600 mL), which was connected to a nitrogen-filled balloon. The vessel was then immersed in water cooled to 4–6 °C with a water bath. Substrate was fed to each reactor for 6–32 min every hour (except 3, 7, 11 a.m. and p.m.). The organic loading rate (OLR) was calculated based on the substrate consumed between two consecutive fillings of the substrate vessel. Increasing the feeding time on consecutive feeding occasions gradually increased the OLR. The initial OLR was of 1–2 g COD per L per day and was increased to 5–6 over the first 43 days. During the first 53 days, the four reactors were run with CH-1. Micronutrients were added as described below. On day 54, the feed to two reactors was changed to a combined hydrolysate-DFE (CH-DFE) consisting of 77% effluent from dark fermentation of WSH-1 (see “Fermentor set-up for dark fermentation”), 7.6% LH and 15.4% deionised water, based on wet weight (see Additional file 1: Table S1, for content of COD and nutrients). The substrate ratio in CH-DFE corresponded to the same ratio of initial raw material DM as for CH-1. The other two reactors were continually fed with CH-1. The pH of each substrate mix was adjusted to pH 7.00 with NaOH (194 g/L), after which deionised water was added to achieve the desired dilution. From day 63, the feeding was increased to 5.5–7.5 g/L/day. Feeding was stopped on day 77, and the temperature was decreased stepwise to room temperature over a few days before reactor liquid circulation also was stopped. On day 124, the temperature was increased stepwise to 37 °C, and feeding was started on day 130 at an OLR of COD 2 g/L/day, which was increased stepwise to a COD of 9 g/L/day on day 188. On day 194, the OLR of one reactor with CH-DFE was increased to COD 12 g/L/day, and the OLR of the other one was kept constant at 9 g/L/day. The experiment was terminated after 217 days. If the pH decreased below 6.8, feeding of that reactor was stopped until the pH increased again. Effluent was removed from the top of each reactor simultaneously with feeding at the bottom and was collected in 2 L cylinders, which were flushed with nitrogen before use. The cylinders were attached to balloons during effluent collection. Liquid was periodically returned manually from the effluent cylinder to the reactors to compensate for volume decrease due to gas production and evaporation. The pH, COD and volatile fatty acid concentrations were measured 30 min after the end of a feeding period.

### Inoculum for anaerobic digestion

Each UASB reactor was inoculated with 300 g wet microbial anaerobic digestion granules and 800 mL effluent from wastewater treatment. Half the granules (150 g) [total solids (TS) content 8.4%] originated from an internal circulation anaerobic digestion reactor treating wastewater (in Falkenberg, Sweden) from beer and soft drink production, operated by Vatten & Miljö i Väst AB (Falkenberg, Sweden). The full-scale reactor was operated at a low ammonium concentration (3–5 mg/L in effluent), an OLR of COD 2–23 g/L/day, at a temperature of 28–35 °C and a hydraulic retention time (HRT) of 0.13–1.3 days (Peter Einarsson and Urban Östman, Vatten & Miljö i Väst AB, personal communication). The other 150 g (TS content 7.7%) granules originated from the same plant, but had been used for another experiment with WSH for 8 months. The elemental composition of the granules from the full-scale reactor was determined. The granules were decanted prior to drying at 105 °C.

### Nutrient demand and nutrient addition in anaerobic digestion

Five percent of the carbon in the substrates was assumed to be assimilated in microbial biomass (net microbial biomass after turnover), based on the data for a non-granular process presented by McCarty [52] (Additional file 1: Table S1). The net assimilation of other elements in microbial biomass was predicted according to Eq. 1. Prediction of concentrations of elements in effluents was calculated according to Eq. 2. Mass loss to the gas phase was ignored.

1 $$\left[ a \right] = \left[ b \right]*0.05*\left[ c \right]/\left[ d \right]$$

2 $$\left[ e \right] = \left[ f \right] - \left[ a \right]$$

where [] is the concentration as mass per volume, *a* is the predicted demand of element for microbial biomass, *b* is the carbon in substrate, *c* is the element in microbial granules, *d* is the carbon in microbial granules, *e* is the element in effluent after centrifugation, and *f* is the element in substrate.

When the nutrients in the feed were predicted to be insufficient, extra salts were added to the combined hydrolysates: 0.1 mg/L Cu as CuCl_2_; 0.04 mg/L Mo as NaMoO_4_·2H_2_O; 0.056 mg/L W as NaWO_4_·2H_2_O and 1.0 mg/L Zn as ZnCl_2_, (Table 2, row P). Additions of Co, Fe, Ni and Se were based on available recommendations for the facilitation of high OLR. Co, Fe and Ni were added according to the levels given by [53]: 0.15 mg/L Co (minimum 0.1–0.2) added as CoCl_2_·6H_2_O; 1 mg/L Fe as FeCl_3_ and 0.2 mg/L Ni as NiCl_2_·6H_2_O. Se (0.16 mg/L) was added as NaSeO_3_, based on a study by Banks et al. [54] On days 167–180, FeCl_3_ was mistakenly added instead of FeCl_2_, as was used by Takashima and Speece [53] and in several other media for anaerobic culture. FeCl_3_ was continually used since it was the form used in the full-scale reactor where inoculum was collected and to avoid more changes. The trace elements were added directly to the reactors, based on recommendations by Takashima and Speece [53]. Trace elements were added twice per week as three 200× solutions: one containing Fe, one containing Cu and Zn, and one containing all other elements.

**Table 2:** Composition of the 6 different steam-pretreated lucerne samples

Pretreated material	1	2	3	4	5	6
DM (%)	15.7	13.4	12.9	12.9	14.1	15.0
WIS (%)	9.5	7.8	7.3	6.9	8.6	8.7
Composition of the WIS (in % of WIS)						
Glucan	41.5	41.2	41.3	40.7	42.9	40.4
Xylan	7.4	6.9	6.5	6.3	7.3	5.9
Galactan	2.0	2.0	2.6	2.9	1.5	1.5
Arabinan	1.8	1.6	1.7	1.8	1.9	1.7
AIL	36.4	40.2	36.0	37.2	35.5	37.3
ASL	1.5	1.7	1.5	1.4	1.4	1.4
Ash	1.2	0.5	1.2	1.2	0.7	2.1
Total (%)	91.8	94.1	90.7	91.5	91.3	90.3
Composition of the liquid fraction (in g/L)						
Glucose*	0.8	0.6	0.6	0.8	0.8	0.9
Xylose*	7.8	6.9	7.2	7.5	7.2	8.2
Galactose*	2.4	1.5	1.6	2.5	2.4	2.0
Arabinose*	2.0	1.3	1.5	1.9	1.8	1.7
Lactic acid	7.3	7.1	6.8	7.0	6.7	7.7
Acetic acid	7.9	7.6	7.3	10.3	9.7	11.6

*AIL* acid insoluble lignin, *ASL* acid soluble lignin

* Sum of monomers and oligomers, expressed as monomer concentration

### Analytical methods

The compositions of the steam-pretreated materials, samples from EH and samples from dark fermentation were analysed using two HPLC instruments (Waters HPLC system, Milford, MA, US and Jasco Co., Tokyo, Japan), each equipped with a refractive index detector (Shimadzu Corp., Kyoto, Japan and Erc Inc., Huntsville, AL, USA, respectively). Glucose, xylose, galactose, arabinose and mannose were separated using an Aminex HPX-87P column (Bio-Rad, Hercules, CA, USA) at 85 °C; alternatively cellobiose, glucose, xylose and arabinose were separated in two Shodex SP-0810 Columns (Shodex Japan) in series. A flow rate of 0.6 mL/min with water as eluent was used in both protocols. Acetic acid, butyric acid, ethanol, formic acid, furfural, galacturonic acid, glucuronic acid, hydroxymethylfurfural (HMF), lactic acid, propionic acid, 2-propanol, succinic acid and valeric acid were separated on an Aminex HPX-87H column (Bio-Rad, Hercules, CA, USA) at 60 °C with a flow rate of 0.6 mL/min using 5 mM sulphuric acid as eluent.

The optical density was determined at 620 nm using an Ultraspec 2100 pro spectrophotometer (Amersham Biosciences, United Kingdom). COD was measured with LCK114 test kits (HACH, Loveland, CO, USA). TS, equally DM after drying at 105 °C, and VS were determined using standard methods [55]. The DM content of the silage was corrected for volatile compounds, according to correction factors developed for grass silage [56]. Partial alkalinity (titration to 5.75), total alkalinity (titration to 4.3) and bicarbonate alkalinity were determined according to Jenkins et al. [57].

CO_2_ and H_2_ were quantified using a gas chromatography with a dual-channel Micro-GC (CP-4900; Varian, Middelburg, The Netherlands), as described previously [58]. Methane volume was measured continuously with an automatic methane potential test system (AMPTS), version 2 (Bioprocess Control AB, Lund, Sweden). The temperature was measured by the AMPTS and the software expresses the gas volume as dry gas at 0 °C and surrounding pressure. The biogas was stripped from carbon dioxide prior to the AMPTS in a similar manner as described in the AMPTS protocol (Bioprocess control AB) but with larger traps: 2 one-litre bottles, serially connected, containing 2*800 mL of 3 M NaOH and thymolphthalein pH indicator.

Fermentation effluents were centrifuged at 10,000×*g* for 10 min or 5000×*g* for 20 min in a Sorvall Lynx 4000 centrifuge (Thermo Fisher Scientific Inc., Waltham, MA, USA) prior to elemental analysis of the supernatant. Solid samples were dried at 105 °C prior to further sample preparation for elemental analysis. Crops were finely ground and mixed after drying. The elements B, Ca, Cu, Fe, K, Mg, Mn, Na, P and S were analysed with inductively coupled plasma optical emission spectroscopy (ICP-OES) (Optima 8300, PerkinElmer Inc., Waltham, MA, USA), while Co, Cr, Mo, Ni, Se, Zn and W were analysed with inductively coupled plasma mass spectroscopy (ICP-MS) (Aurora Elite, Bruker Corporation, Bremen, Germany). The measurement uncertainty for both ICP methods was 0.2–5%, depending on the element. Total carbon and total nitrogen in liquid samples were analysed with a total organic carbon analyser coupled to a total nitrogen measurement unit (Shimadzu Corp., Kyoto, Japan), and in solid samples with a Vario MAX CN element analyser (Elementar Analysensysteme GmbH, Langenselbold, Germany). The measurement uncertainty was 1–2%. Phosphate was analysed on an 861 Advanced Compact IC (Metrohm AG, Herisau, Schweiz), with a measurement uncertainty of 1–2%. Ammonium was analysed with a flow injection analyser (Foss A/S, Hilleroed, Denmark).

## Results and discussion

### Composition of steam-pretreated wheat straw hydrolysate

The composition of raw and steam-pretreated wheat straw has been determined previously by Bondesson and Galbe [45]. EH of steam-pretreated wheat straw at 10% WIS in the present study resulted in a hydrolysate containing 57 ± 3 g/L glucose and 31 ± 3.5 g/L xylose, which correspond to a sugar yield of approximately 0.5 g sugar per g DM raw wheat straw, which is above 90% of the theoretical yield according to data found in Bondesson and Galbe [45]. Hydroxymethylfurfural (HMF) and furfural concentrations were measured but were below detectable limits (< 0.25 g/L).

### Composition of steam-pretreated lucerne samples

Although acetic acid impregnation prior to steam pretreatment of wheat straw has been investigated previously in the production of bioethanol [45] and combined bioethanol and biogas production [43], steam pretreatment of ensiled lucerne has, to the best of the authors’ knowledge, not been studied before. The solid fraction of steam-pretreated lucerne samples consisted of 40.4–42.9% glucan, 5.9–7.4% xylan, and 36.9–41.9% lignin (Table 2). The pH of the liquid fractions was between 4.9 and 5.2, and they contained high amounts of lactic acid (6.7–7.3 g/L) and acetic acid (7.3–11.6 g/L), due to high concentration of these acids in the ensiled material. The glucose and xylose contents (expressed as monomer concentration) in the liquid fractions were 0.6–0.9 and 6.9–8.2 g/L, respectively (Table 2). Generally, there were only very slight differences between the compositions of the various pretreated lucerne materials. The rather high residual xylan content in the solid fractions after pretreatment together with a relatively low concentration of xylose in the liquid fractions (Table 2) indicated that the severity of the pretreatment was low. This was also supported by the observation that after steam pretreatment the concentrations of HMF and furfural were below the detection limit. Furthermore, the high pH after pretreatment indicated that the material contained compounds that had a buffering effect during the process, which might also have contributed to the low severity. High pH and low furan contents in hydrothermally pretreated lucerne have also been reported previously [42].

### Enzymatic hydrolysis of steam-pretreated lucerne samples

**Fig. 2:**
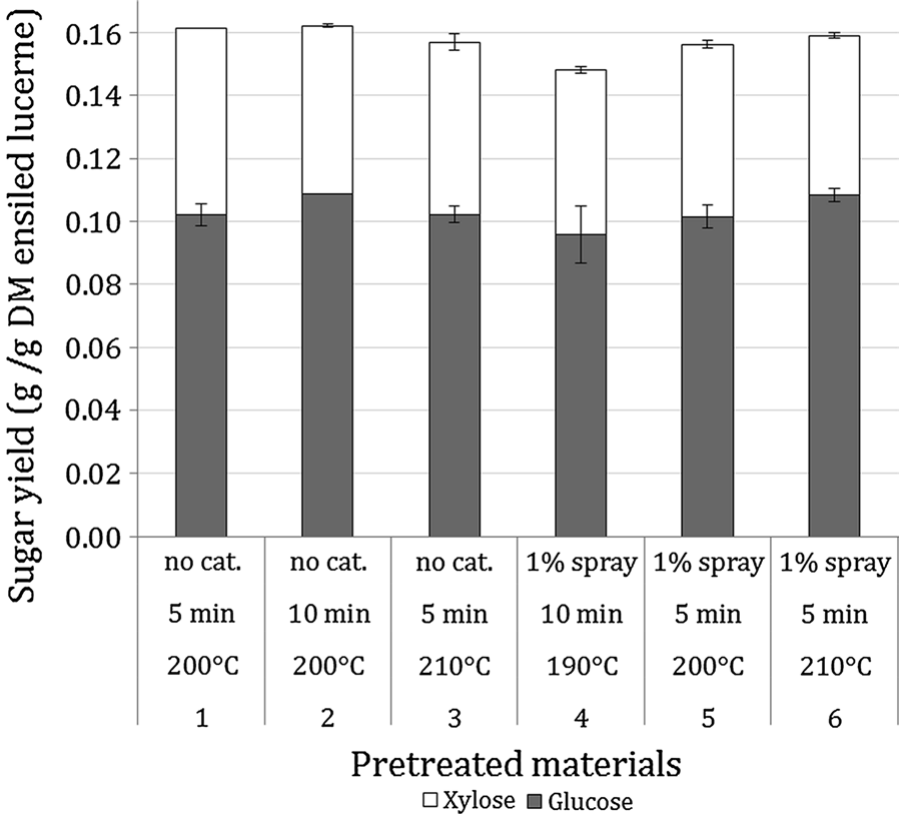
Glucose and xylose yields obtained after steam pretreatment of ensiled lucerne at six different conditions, and after 72 h of EH of the pretreated materials at 5% WIS content using Cellic Ctec2 enzymes at 10  FPU/g WIS loading at 45 °C and pH 4.8

To identify the best pretreatment conditions for the lucerne silage, the hydrolysability of the 6 pretreated materials was investigated. After 72 h of EH at 5% WIS, the glucose and xylose concentrations in the hydrolysates were 9.9–11.7 and 5.3–6.4 g/L, respectively. These sugar concentrations corresponded to overall yields of the sum of glucose and xylose of 0.148–0.162 g per g DM initial ensiled lucerne. The highest overall yield was obtained in pretreatment 2 (Fig. 2), which was 74.9% of the theoretical yield based on the glucose and xylose content of ensiled lucerne. Galactose and arabinose concentrations in the EH were under 0.5 g/L and, therefore, these sugars were neglected in the calculations. Based on these results, pretreatment 2 (200 °C, 10 min, no catalyst) was selected for the preparation of the LH for the fermentation experiments. The sugar concentrations in the larger batch hydrolysed at 7.5% WIS were 12.9 g/L glucose and 4.8 g/L xylose, corresponding to a sugar yield of 0.12 g glucose and xylose per g DM initial ensiled lucerne. This yield was lower than that obtained in the smaller batch at 5% WIS.

Interestingly, the concentration of COD was very similar between WSH and LH (107 and 112 g/L, respectively (Additional file 1: Table S1) despite the lower WIS content applied to LH in EH and the lower sugar yield of LH. Overall the COD yield per kg initial DM was 0.61 kg for WS and 0.76 kg for LH. The higher COD yield from LH can be explained by solubilisation of proteins and other compounds in LH. As expected, the concentrations of nitrogen and phosphorous in LH were considerably higher (20 and 6 times, respectively) than in WSH (Additional file 1: Table S1, rows C and D). Moreover, the contents of Ca, Mg, S, B, Mo and Se were 24, 15, 7, 28, 12 and 333 times higher in LH than in WSH.

The addition of 1 wt% acetic acid was found to be unnecessary prior to steam pretreatment as ensiled lucerne contained significant amounts of acetic acid from the ensiling process and, moreover, similar sugar yields were obtained regardless of the conditions applied. This exemplifies that ensiled materials can reduce the cost of chemicals in steam pretreatment. In a previous study, ensiling of wheat straw prior to hydrothermal treatment increased the effect of pretreatment, thereby enabling to reduce the pretreatment temperature [59].

Pretreatment of the lucerne used in the current study at the same conditions as optimal for wheat straw (pretreatment 4) would give similar sugar yield as the chosen condition did (pretreatment 2). Co-pretreatment of intercropped and co-harvested lucerne and wheat straw can, therefore, likely be feasible. However, harvest of lucerne at another maturation stage can influence the effect of pretreatment. Likewise, the use of straw from spring wheat instead of winter wheat as well as the use of other cultivars or cultivation conditions may need different pretreatment conditions.

### Medium and strain adjustment for dark fermentation

A new medium (EB-1), based on the quantities of various elements in the cell mass of *Caldicellulosiruptor*, was designed to optimise the concentration of elements (Additional file 1: Table S1). Specifically, this optimisation was performed to reduce the excess quantities of the elements phosphorus, magnesium, molybdenum, nickel and sulphur, while the concentrations of copper, iron and zinc were increased compared to those in the modified SL-10 medium [36], to avoid nutrient limitation. Yeast extract was completely omitted from the EB-1 medium. The phosphorus concentrations of the medium were decreased by 80% (94% per g sugar) compared to the modified DSM640 medium previously used by Zeidan and van Niel [58]. According to Ljunggren and Zacchi [25], replacing or reducing nutrients, including yeast extract and phosphate, may amount to lowering the total costs of the process by 28–50%.

Osmotolerant strains were selected as they allow the use of more concentrated hydrolysates and, therefore, reduce the water usage. It has previously been demonstrated that *C. saccharolyticus* wild-type strain is completely inhibited when cultured on a 20% mixture of WSH [18]. Synthetic co-cultures of *C. saccharolyticus* and *C. owensensis* wild types have been previously demonstrated to allow an increased formation of biofilm, thereby allowing for cell mass retention, increased sugar consumption and hydrogen productivity rates [60]. Therefore, osmotolerant strains of *C. saccharolyticus* and *C. owensensis* were cultivated together in co-culture on a defined medium containing EB-1, modified DSM 640 medium, WSH-1 or CH-1.

In each medium, a higher amount of xylose was consumed compared to glucose even though glucose was present at a higher concentration (Table 3). This is consistent with van de Werken et al. [61] who showed *Caldicellulosiruptor* has a preference for xylose over glucose. The quantity of cell mass is lower when cultivated on EB-1 trace elements compared to the DSM 640 medium. A significant increase in cell mass was observed with the addition of either CH-1 or WSH-1 (Table 3). The trace element concentrations are higher in hydrolysates than in defined media due to the contribution from the hydrolysates on top of the added trace elements that was equal to all media investigated (Additional file 1: Table S1). The lower cell mass obtained with EB-1 than with DSM 640 could be caused by elemental metal deficiencies in EB-1. Therefore, further optimisation of this medium is required.

**Table 3:** Substrate and products concentrations of osmotolerant *Caldicellulosiruptor* cultivation on hydrolysates and defined media EB1 and modified DSM 640

Component (g/L)	CH-1		WSH-1		EB-1		Modified DSM 640	
	Before DF	After DF	Before DF	After DF	Before DF	After DF	Before DF	After DF
Microbial biomass		1.13 ± 0.00		1.34 ± 0.01		0.56 ± 0.04		0.72 ± 0.02
Glucose	14.52	10.81 ± 0.45	18.82 ± 0.05	14.84 ± 0.17	18.82	18.17 ± 0.56	18.82	18.47 ± 0.47
Xylose	6.25	1.55 ± 0.18	8.19 ± 0.24	1.914 ± 0.28	8.2	3.50 ± 0.21	8.2	2.84 ± 0.93
Arabinose	0.42	ND	0.58 ± 0.01	ND	0.59	ND	0.59	ND
Acetate	1.79	7.74 ± 0.36	1.60 ± 0.56	7.20 ± 0.56	ND	2.80 ± 0.38	ND	3.15 ± 0.45
Lactate	0.39	0.61 ± 0.075	ND	0.18 ± 0.11	ND	0.27 ± 0.07	ND	0.20
Ethanol	0.06	0.12 ± 0.000	ND	0.82 ± 0.02	ND	0.15 ± 0.01	ND	0.11 ± 0.02
Propionate	0.095	0.18 ± 0.030	ND	ND	ND	ND	ND	ND

*ND* not detected

### Dark fermentation with osmotolerant *Caldicellulosiruptor* on different forms of hydrolysate

**Fig. 3:**
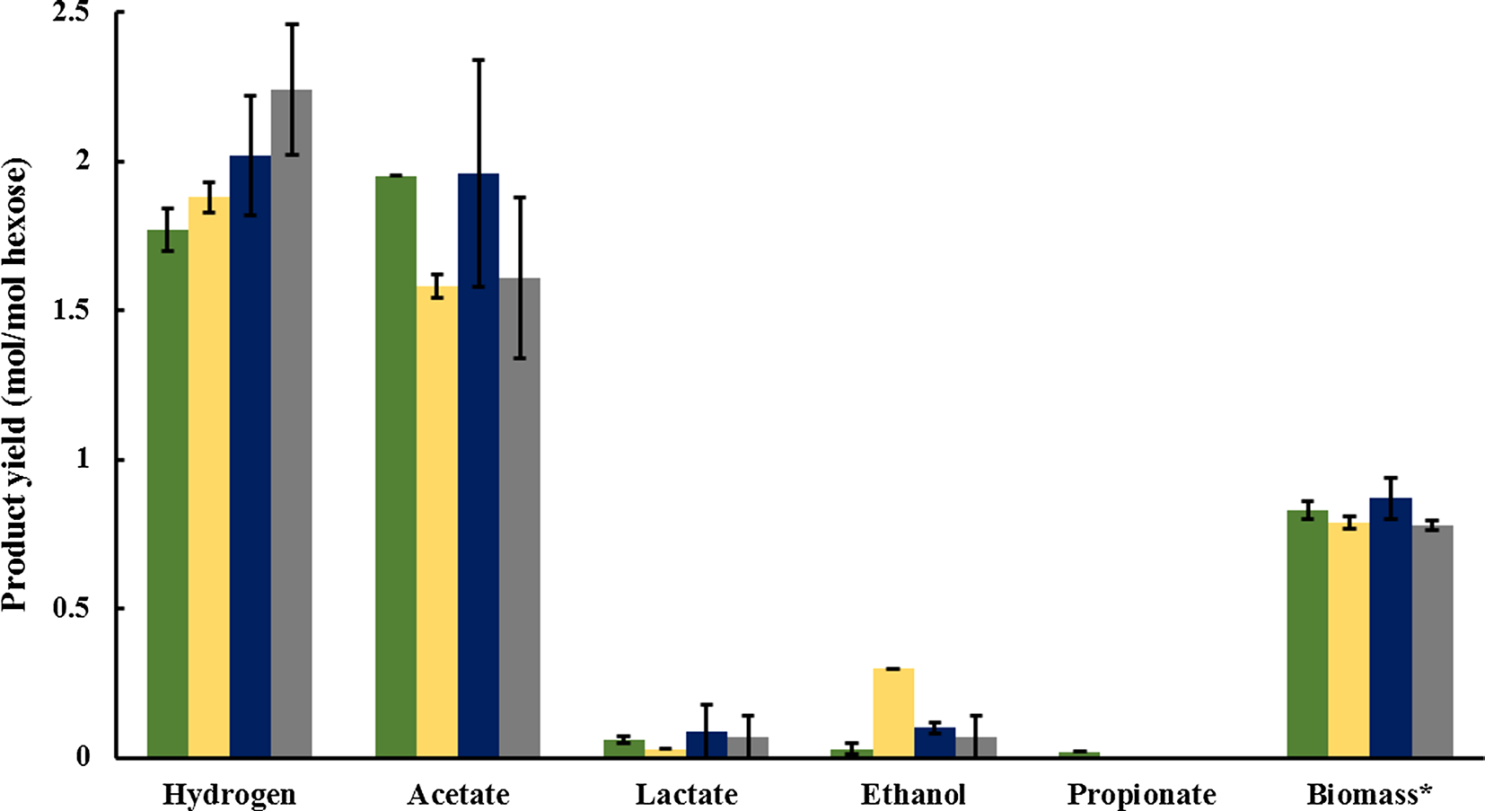
Product yields of osmotolerant *Caldicellulosiruptor* cultivation on hydrolysates and a defined medium EB1-TE. Yields shown are given for cultivation of osmotolerant *C. saccharolyticus* and *C. owensensis* on CH-1 (green), WSH-1 (yellow), EB-1 (blue) and modified DSM 640 (grey). All yields are presented in mol/mol hexose equivalent. Hexose equivalent, which are calculated by dividing the total sugar concentration (both hexoses and pentoses; g/L) by 180.1 g/mol. *Biomass values are given as C-mol/mol

The osmotolerant strains cultivated on both defined media and hydrolysates showed similar product yields to each other with the exception of WSH-1, which displayed a significantly increased yield of ethanol compared to any of the other media (Fig. 3). However, each cultivation had a relatively low yield of hydrogen of between 1.8 and 2.3 mol H_2_/mol hexose (Fig. 3) compared to the yield of 3.4 mol H_2_/mol hexose previously described for wild-type *C. saccharolyticus* on wheat straw hydrolysate [18] and 3.8 mol H_2_/mol for wild-type *C. owensensis* on a modified DSM 640 medium [51]. In contrast, the yield of acetate in each medium composition approached the theoretical maximum of 2 mol/mol hexose (Fig. 3). A reason for the hydrogen per acetate ratio being substantially below a value of two might be a result of osmotic stress on the cell. Osmotic pressure has been previously shown to influence activity of Ni–Fe hydrogenases in *E. coli*, though in that case, hydrogenase activity was reduced by low osmolality [62]. Although yields of 1.8 mol H_2_/mol hexose have been described with wild-type *C. bescii*, this is due to the equimolar production of acetate and lactate [63]. In this study, however, only trace amounts of lactate were detected. However, even though hydrogen yield is lower than for the wild type, the redox balance depicts no missing electrons (Table 4). This could be due to a higher yield of biomass.

**Table 4:** Hydrogen concentration in the gas phase, and the carbon and redox balances of osmotolerant *Caldicellulosiruptor* strains cultivation on hydrolysates and defined media EB1 and modified DSM 640

	CH-1	WSH-1^a^	EB-1	Modified DSM 640
H_2_ (%)	1.71 ± 0.10	3.86 ± 0.04	0.99 ± 0.22	1.47 ± 0.17
% Carbon balance	96.9 ± 7.3	96.8 ± 1.4	101.4 ± 0.9	99.4 ± 5.4
% Redox balance	97.5 ± 7.6	97.8 ± 3.2	100.7 ± 0.0	96.6 ± 5.3

^a^Total volume of WSH-1 was 1.5 L, all other fermentations were conducted with a total volume of 1 L

### Anaerobic digestion

High and stable COD removal and a high methane yield were obtained during anaerobic digestion of CH-DFE at an average OLR of COD 5.4 g/L/day for a period of three times the HRT (Table 5). One of the reactors fed with CH-1 also operated with high and stable COD removal and high methane yield for a period of three times the HRT at this average OLR (Table 5). However, the liquid of one of the two reactors fed with CH-1 got acidic after a period of 2.3 times the HRT, when the OLR temporarily exceeded COD 7 g/L/day (Table 5). Likewise, the liquid of the other reactor fed with CH-1 also acidified later after a period with an OLR exceeding COD 7 g/L/day. Stable and high methane yield was demonstrated at a higher OLR (COD 8.5 g/L/day) over a period of three times the HRT for both reactors fed with CH-DFE. In one of the reactors fed with CH-DFE, the reactor liquid acidified later when the OLR was temporarily increased to COD 12 g/L/day, while the other one operated stably at sustained OLR of COD 8–9 g/L/day until the experiment was ended.

**Table 5:** Operational variables for the AD experiments in UASB reactors

	CH-1, lower OLR	CH-DFE, lower OLR	CH-DFE, higher OLR
OLR, COD (g/L/day)	5.4 ± 0.5	5.4 ± 0.5	8.5 ± 0.2
HRT (day)	5.9 ± 0.3	5.5 ± 0.5	3.8 ± 1.3
Duration (day)	17.0 ± 2.0	19 ± 0	13.3 ± 4
Methane yield per COD fed, (mL/g)	268 ± 7	262 ± 17	265 ± 15
Methane yield per COD fed, % of maximum	76.7 ± 2.2	74.8 ± 4.7	75.6 ± 4.3
Effluent COD (g/L)^a^	3.6^b^	3.8 ± 0.3	5.1 ± 0.6
COD removal (%)^a^	89.2^b^	88.7 ± 5.9	84.8 ± 1.9
Effluent pH^a^	6.84^b^	7.22 ± 0.02	7.20 ± 0.03
Effluent acetic acid^a^ (g/L)	0.11^b^	0.21 ± 0.07	0.23 ± 0.13
Effluent propionic acid^a^ (g/L)	0.11^b^	0.05 ± 0.07	0.27 ± 0.14
Effluent fatty acids, total^a, c^ (g/L)	0.22^b^	0.26 ± 0.14	0.60 ± 0.26
Partial alkalinity^a^ in effluent (g/L)	1.2^b^	2.5 ± 0.0	1.8 ± 0.2
Total alkalinity^a^ in effluent (g/L)	1.3^b^	3.0 ± 0.1	2.7 ± 0.0

*COD* chemical oxygen demand, *HRT* hydraulic retention time, *OLR* organic loading rate

^a^After running the experiment for a duration corresponding to three times the HRT

^b^Value for one reactor, data missing for the other

^c^Volatile fatty acids with 1–5 carbons and lactic acid

When using CH-1, the partial alkalinity of the anaerobic digestion effluent was only 1.2 g/L. When using CH-DFE, to which buffering agents were added prior to dark fermentation, the partial alkalinity of the anaerobic digestion effluent was higher, but still low: 1.8–2.5 g/L (Table 5). This is a low alkalinity for UASB reactors according to [64] and makes the processes sensitive to disturbances. Another weakness in the process design was that the effluent was pumped out at the same rate as the feed was pumped in, which sometimes caused the liquid level to decrease below the level for recirculation outflow, since some mass is lost to the gas phase. Spontaneous outflow using a water-lock or other solutions that ensure an adequate liquid level should have been applied. When recirculation is interrupted, the OLR will increase in the lower part of the reactors and acids will easily accumulate [64], which is a likely reason for the acidification seen. The chance that sugar-derived inhibitors HMF and furfural were the cause of acidification might be small as in the current study the concentrations of these inhibitors were lower than the concentrations (2 g/L and 0.8 g/L, respectively) shown to cause inhibition [65]. Other potential inhibitors, such as lignin-derived inhibitors [13], were not analysed.

The methane yield and COD conversion using CH-DFE in the present study were not significantly different to those reported by Pawar et al. [18] when using the effluent from dark fermentation of WSH with half of the COD concentration. Also, the methane yield was not significantly different from that presented in a study using hydrothermally pretreated wheat straw for anaerobic digestion in UASB reactors at 55 °C [66], but at a lower OLR (2.8 g_COD_/L/day) with highly diluted substrate (COD 3.8 g/L compared to 34 g/L in the present study). However, Kaparaju et al. [66] found that increasing the OLR to COD 7 g/L/day (by increasing the substrate concentration to COD 9.5 g/L), for unknown reasons, drastically reduced the methane yield to 174 mL/g_COD_. The OLRs for the hydrolysates demonstrated in the current study and by Pawar et al. [18] are higher than typical for anaerobic digestion of chopped energy crops in completely stirred tank reactors, which is the dominant procedure. For instance, Nges and Björnsson [67] demonstrated decreasing methane yields for three crops mixtures when increasing the OLR above 3–4.5 kg VS per m^3^ reactor and day.

### Lucerne as nutrient provider in anaerobic digestion

It was predicted that combining LH, corresponding to 24% of the COD, with WSH (76% of COD), which are the proportions in CH-1, would satisfy the demand for N and P as well as for B, Ca, K, Mg, Mn, Na and Se in anaerobic digestion, based on the assumptions described in “Nutrient demand and nutrient addition in anaerobic digestion”. Likewise, it was found that the substrate proportions in CH-DFE (same proportions as in CH-1 based on initial raw material) would satisfy the demand of the same nutrients. The amounts of several micronutrients were, however, predicted to be insufficient in the combined hydrolysates and were added together with extra selenium at levels based on practical trials by Banks et al. [54]. The calculated nutrient demands was also compared to the concentrations reported by Takashima and Speece [53] to be necessary to support the conversion of 30 g acetate/L/day to methane. The modelled iron and sulphur demands were much higher than the concentrations applied by Takashima and Speece [53] and also higher than the content reported for cultures containing only methanogens [68]. This could be the result of iron sulphide precipitation on the analysed anaerobic digestion granules rather than the microbial demand for these elements. The sulphur and iron demand was, therefore, based on the levels used by Takashima and Speece [53] rather than the carbon to nutrient quotes in the analysed granular biomass.

The concentrations of macronutrients predicted (based on the assumption of assimilation of nutrients as described in “Nutrient demand and nutrient addition in anaerobic digestion”) and measured in the effluent from anaerobic digestion of CH-1 complied well, with maximum of 15% deviation for most elements (Additional file 1: Table S1, row O and Q). Fe and S deviated, as predicted, with higher effluent concentrations than predicted and also Na deviated. The nutrients supplied by combining LH and WSH at the shares of 24% and 76% of COD, respectively, in CH-1 (Additional file 1: Table S1, row M), corresponding to 18% lucerne of initial DM of the raw materials, could support an OLR of COD 5.4 g/L/day and 89% COD removal (Table 5). A higher share of lucerne has been found when intercropping lucerne and wheat [32]. This suggests that it will probably be possible to use intercropped lucerne and wheat straw for anaerobic digestion without addition of macronutrients. However, composition of the crops can vary with harvest time and cultivation conditions and further experiments are needed to prove this assumption.

Although the macronutrients supplied by mixing LH and WSH supported a high COD conversion, more nutrients might be needed to ensure long-term stability or at start-up of a granular process from non-granular sludge. A comparison of the nutrient concentrations in the effluents to general recommendations for excess concentrations of different nutrients in anaerobic digestion of waste water [69] and recommendations for concentrations in the feed [70] indicates that both the processes fed with CH-1 and CH-DFE could benefit from more ammonia–nitrogen, Ca, Co, Cr, Cu, Fe, Mg, Mn, Mo and W and also more phosphate for CH-1 (Additional file 1: Table S1, row Q, U, V and X compared to row Y). However, the lowest ammonia concentration found in the effluents (5 mg/L, Additional file 1: Table S1) is similar to that in the full-scale reactor from where the inoculum was collected (3–5 mg/L), which could support an OLR of at least COD 12 g/L/day. Based on Yu et al. [71] and Yu et al. [72], the calcium and iron levels in the effluents in the current study are probably sufficient to not limit degradation of organic matter, since granular sludge was added as inoculum. However, at the start-up of a granular process a larger amount of Ca^2+^ than used in the current study is recommended [71] and a larger amount of Fe^2+^ might [73] or might not [72] be needed. Schmidt and Ahring [74] report that as little as 12 mg/L of Mg^2+^ in the feed (compared to 22–27 mg/L in the current study) could support 97% acetate conversion in UASB reactors loaded with acetate 8 g COD per day. However, some microbial biomass was lost during the operation, indicating that higher Mg^2+^ levels are likely needed for stable long-term operation. 240 and 740 mg/L could sustain the biomass level. No elements were found to be present at inhibiting levels in CH-1 and CH-DFE when the data in Additional file 1: Table S1 were compared with the data summarised by Romero-Güiza et al. [70].

### Energy yield per initial raw material

In addition to the high conversion of organic material in the hydrolysates, found in the current study, utilisation of the solid side streams (Fig. 1) would be needed to reach high substrate conversion efficiency. The methane yield of the CH-DFE corresponds to 4.32 MJ per kg initial DM, based on the lower heating value (LHV) of methane. The hydrogen energy yield from the WSH used for the production of CH-DFE to 0.18 MJ (LHV) per kg initial DM. Methane and hydrogen sum up to 4.5 MJ per kg initial DM. Nkemka and Murto [14] report an LHV of untreated wheat straw of 16.3 MJ per kg DM. Approximating the LHV for dry lucerne to be the same as for dry wheat straw the energy yield would be only 28% of the total raw material, despite a yield of methane and hydrogen corresponding to more than 75% of the COD in the hydrolysates (Fig. 3 and Table 5). A large share of the raw material can be found in the solid side streams. Nkemka and Murto [14] report a methane energy yield of 2.5 MJ (LHV) methane, or 15% of the energy in the initial raw material, per kg initial DM of wheat straw from the solid fraction after EH (when subjected to batch mode anaerobic digestion). The solid fraction (rich in lignin) can be used for multiple purposes. Barta et al. [17] showed through modelling that using the solid fraction of steam-pretreated and enzymatically saccharified hemp for combined heat and power production resulted in self-supporting and excess production of heat and electricity, in combined ethanol and biogas production. Similar results were found for biohythane process [75], but in that case some of the energy was needed for compressing gas for stripping the biohydrogen reactor.

Comparing the methane energy yield from lucerne and wheat straw in other studies further illustrates the importance of valorising the solid side streams for an overall high substrate utilisation. The methane energy yield from CH-1 is lower than that from non-pretreated lucerne, subjected to batch mode anaerobic digestion, reported by Hakl et al. [27]: 6.4–10.0 MJ as LHV, per kg DM non-pretreated material. The methane energy yield from pretreated wheat straw without enzymatic hydrolysis and subsequent fractionation, subjected to batch mode anaerobic digestion, was 10.7 MJ (LHV) per kg initial DM, corresponding to 66% of the energy in the raw material.

### Future studies

The current study showed that dark fermentation of WSH at a sugar concentration of 30 g/L was possible. However, the hydrogen yield and productivity were lower when a sugar concentration of 10 g/L was used as reported by [18]. In future studies, it would be interesting to explore the productivity and yield in dark fermentation when exchanging part of the water used to dilute the feed to dark fermentation with effluent from the anaerobic digestion process. Possibly the water and energy demand could be decreased by this approach, without decreasing the hydrogen productivity and yield from the levels demonstrated by Pawar et al. [18]. Recycling of effluent is common in anaerobic digestion and has been used to provide alkalinity and to dilute incoming feeds [64]. Modelling of recycling from a mesophilic photofermentation step to a 70 °C dark fermentation step has demonstrated the potential of reducing the water and energy demand by up to 90% [22]. Recycling of liquid from a mesophilic UASB to a 55 °C dark fermentation step using starch wastewater was shown to decrease the need for the addition of base to the dark fermentation reactor with 88% [76]. It remains to be investigated whether the accumulation of inhibitors would restrict such recycling of effluent from anaerobic digestion of lignocellulosic substrates.

As demonstrated in the current study, the liquefaction of solid plant biomass does allow the use of high rate UASB reactors in anaerobic digestion. As shown by Barta et al. [17], the reactor cost can be reduced compared to that of a completely stirred tank reactor due to higher OLR and thereby smaller reactor volume. However, liquefaction of solid plant biomass by steam pretreatment and enzymatic hydrolysis with external enzymes is also costly [17]. Equalling many mixed anaerobic digestion cultures, the genus *Caldicellulosiruptor* has the potential to directly degrade cellulose and hemicellulose [77, 78]. It would be of interest to compare the performance of the current process setup to a process fed with steam-pretreated wheat straw and lucerne to dark fermentation, without an enzymatic pretreatment step. This would also reduce the osmolarity due to the presence of polymeric rather than monomeric sugars and could thereby potentially allow higher substrate concentrations.

## Conclusions

This study demonstrates the possibility of reducing the water addition to WSH by 26% and the phosphorus additions by 80% and the omission of yeast extract in dark fermentation with *Caldicellulosiruptor* species, compared to previous reports. WSH and combined WSH and LH were well tolerated by osmotolerant co-cultures. The yield was not significantly different when using defined media or hydrolysates with the same concentrations of sugars. The sugar concentration was negatively correlated with the hydrogen yield though, when comparing the results to previous studies.

Combined WSH and LH and effluent from dark fermentation of WSH plus LH were efficiently converted to methane in anaerobic digestion. Degree of dilution of hydrolysates was defined by the dark fermentation process. The effect of using hydrolysates with less or no water addition, after enzymatic hydrolysis, remains to be explored. For an overall high substrate conversion degree, utilisation of the solid fraction after enzymatic hydrolysis would also be required, in addition to the studied processes.

The macronutrients in combined hydrolysate with 24% LH and 76% WSH, based on COD, could support an efficient conversion of organic matter to methane in anaerobic digestion. For start-up of a granular process and long-term stability, a higher share of LH, or supplements, might be needed.

Finally, the presented results indicate that the same conditions for pretreating wheat can be applied to lucerne with little or no influence on the sugar yield. Consequentially, it will probably be possible to co-pretreat wheat straw and ensiled lucerne in this process. Further experiments are needed to prove this assumption. Intercropping of lucerne and wheat, and harvesting the wheat straw with the lucerne biomass after harvest of the wheat spikes, is an interesting possibility for production of feedstock for conversion to transportation fuel that deserves further attention.

## Additional file

**Additional file 1: Table S1.** Concentration of nutrients in substrates (A to E), microbial biomass DF and AD (F and L), modelled nutrient demand DF and AD (G, N and O), nutrients added to DF (H and I) and to AD (P), DF effluents (J and K), AD effluents (modelled; O and T, and measured; Q, U, V and X) and general recommendations on excess concentrations for AD of wastewaters (Y). Concentrations in AD effluents lower than recommended minimum excess concentrations are red and those equal to or higher than recommended are green. Standard deviations for two separate samples are shown after ±.

## Abbreviations

CDW: cell dry weight
CH-1: combined hydrolysate-1
CH-DFE: combined hydrolysate-dark fermentation effluent
COD: chemical oxygen demand
D: dilution rate
DFE: dark fermentation effluent
DM: dry matter
EH: enzymatic hydrolysis
FPU: filter paper units
HMF: hydroxymethylfurfural
HPLC: high performance liquid chromatography
HRT: hydrolytic retention time
ICP-MS: induced coupled plasma mass spectroscopy
L: lucerne
LH: lucerne hydrolysate
MPR: methane production rate
ND: not detected
OLR: organic loading rate
SP: steam pretreatment
Q_H2_: volumetric hydrogen yield
TS: total solids
UASB: upflow anaerobic sludge blanket
VS: volatile solids
WIS: water insoluble solids
WSH: wheat straw hydrolysate
WSH-1: 33% WSH and 67% water
wt%: percentage by weight
ww: wet weight
